# Prof. Robert Koch’s Method to Cure Tuberculosis

**Published:** 1891-02

**Authors:** 


PROF. ROBERT KOCH'S METHOD TO CURE TUBERCULOSIS. Popularly treated by Dr. Max Birnbaum. Translated by Dr. Fr. Brendecke. Published by H. E. Haferkorn, Milwaukee, Wis. Paper .75, Cloth $1.00.

This is truly a popular treatise, and I think too much so, as it gives all the symptoms of Tuberculosis in its various forms and stages, so that if it were put into the hands of the Laity, it would be very liable to make them think they all had some form of Tuberculosis.

The portion devoted to the prevention of the disease and the cure of those already subject to it could be well put, and to my mind, should be put in every house. A disease which kills more people every year than all others combined should certainly be watched and combatted by all possible means.

Of the success of Koch's treatment it is a little early to speak with any degree of certainty, but it is to be hoped that it may be all that is claimed for it. F. O. S.

---

SYCOSIS.—℞ Iodoform, 4 parts; lanolin, 30 parts. Leache recommends the above to be applied every night, and to be washed off in the morning with hot water.—*Ex.*

---

### CHORDEE.

R Ext. opii aq., 2 grains.
Camph., 4 grains.
M. ft. pil No. II. Sig. One or both on retiring.—*Ex.*

